# Who is our cohort: recruitment, representativeness, baseline risk and retention in the “Watch Me Grow” study?

**DOI:** 10.1186/s12887-016-0582-1

**Published:** 2016-03-24

**Authors:** Susan Woolfenden, Valsamma Eapen, Emma Axelsson, Alexandra Hendry, Bin Jalaludin, Cheryl Dissanayake, Bronwyn Overs, Joseph Descallar, John Eastwood, Stewart Einfeld, Natalie Silove, Kate Short, Deborah Beasley, Rudi Črnčec, Elisabeth Murphy, Katrina Williams, the “Watch Me Grow” study group

**Affiliations:** Sydney Children’s Hospitals Network, Sydney, Australia; University of New South Wales, Sydney, Australia; Academic Unit of Child Psychiatry, South West Sydney Local Health District (AUCS), Sydney, Australia; School of Psychiatry & Ingham Institute, University of New South Wales, Sydney, Australia; Ingham Institute for Applied Medical Research, Liverpool, Australia; Early Years Research Group, Ingham Institute, Sydney South West Local Health District, Sydney, Australia; Epidemiology Group, Healthy People and Places Unit, South Western Sydney Local Health District, Sydney, Australia; School of Public Health and Community Medicine, University of New South Wales, Sydney, Australia; Olga Tennison Autism Research Centre, La Trobe University, Melbourne, Australia; South Western Sydney Clinical School, University of New South Wales, Sydney, Australia; Community Paediatrics, South Western Sydney Local Health District, Sydney, Australia; Centre for Disability Research and Policy, Brain & Mind Research Institute, University of Sydney, Sydney, Australia; Discipline of Paediatrics and Child Health, University of Sydney, Sydney, Australia; Speech Pathology Unit, Liverpool Hospital, Sydney, Australia; NSW Kids and Families (NSW Health), Sydney, Australia; Department of Paediatrics, University of Melbourne, Sydney, Australia; Developmental Medicine, Royal Children’s Hospital, Sydney, Australia; Murdoch Children’s Research Institute, Sydney, Australia

**Keywords:** Participation bias, Recruitment, Birth cohort

## Abstract

**Background:**

The “Watch Me Grow” (WMG) study examines the current developmental surveillance system in South West Sydney. This paper describes the establishment of the study birth cohort, including the recruitment processes, representativeness, follow-up and participants’ baseline risk for future developmental risk.

**Methods:**

Newborn infants and their parents were recruited from two public hospital postnatal wards and through child health nurses during the years 2011–2013. Data was obtained through a detailed participant questionnaire and linked with the participant’s electronic medical record (EMR). Representativeness was determined by Chi-square analyses of the available clinical, psychosocial and sociodemographic EMR data, comparing the WMG participants to eligible non-participants. Reasons for non-participation were also elicited. Participant characteristics were examined in six, 12, and 18-month follow-ups.

**Results:**

The number of infants recruited totalled 2,025, with 50 % of those approached agreeing to participate. Reasons for parents not participating included: lack of interest, being too busy, having plans to relocate, language barriers, participation in other research projects, and privacy concerns. The WMG cohort was broadly representative of the culturally diverse and socially disadvantaged local population from which it was sampled. Of the original 2025 participants enrolled at birth, participants with *PEDS* outcome data available at follow-up were: 792 (39 %) at six months, 649 (32 %) at 12 months, and 565 (28 %) at 18 months. Participants with greater psychosocial risk were less likely to have follow-up outcome data. Almost 40 % of infants in the baseline cohort were exposed to at least two risk factors known to be associated with developmental risk.

**Conclusions:**

The WMG study birth cohort is a valuable resource for health services due to the inclusion of participants from vulnerable populations, despite there being challenges in being able to actively follow-up this population.

## Background

Early detection of developmental disorders and timely intervention has the potential to alter adverse developmental trajectories [1–5]. Unfortunately, up to 70 % of children who have developmental problems are not identified until after they start primary school [3, 6]. Developmental surveillance provides a systematic approach to identifying individuals at risk of having a significant developmental problem, and who could benefit from further assessment and early intervention [1–5]. The key components of such surveillance include ongoing contact with families and children, anticipatory guidance, and promotion of child development through regular monitoring and responding to developmental concerns. This is achieved using parental history, clinical observation and use of a validated surveillance tool over multiple time periods [7, 8]. In the state of New South Wales (NSW), Australia, developmental surveillance is undertaken by child health nurses in Early Childhood Health Clinics and doctors and practice nurses in General Practice. There is evidence from international reviews of current practice in primary health care that developmental surveillance in primary health care is not universal or consistent [9–11].

The “Watch Me Grow” (WMG) study was designed to evaluate the performance of the current developmental surveillance system in accurately identifying children at risk of developmental disorders in South West Sydney by: 1) assessing non-completion of six, 12, and 18-month developmental surveillance at well child checks and associated risk factors; 2) determining the prevalence of moderate or high developmental risk as determined by the *Parents’ Evaluation of Developmental Status PEDS* [12] and associated risk factors at these checks; and 3) ascertaining the accuracy of the current NSW universal developmental surveillance program. The WMG study protocol has been previously reported [13]. A key component of WMG is the establishment of a longitudinal birth cohort. This methodology is essential to examine risk factors for non-completion of six, 12, and 18-month developmental surveillance at well child checks, as well as the prevalence of parental concerns on the *PEDS* indicating moderate or high developmental risk and associated risk factors [12].

Representativeness of a cohort, like the WMG cohort, will influence its ability to answer its research questions, and for its findings to have direct application to health service improvement. Differential study participation, such as higher non-participation rates among more disadvantaged families (including those living in poverty or from minority ethnicities), may lead to an underestimated prevalence of important outcomes in birth cohorts in these high-risk groups, and limit applicability of study findings [14, 15]. A recent systematic review, which included primary studies from Australia, found an increased prevalence of parental concerns indicating high developmental risk on the *PEDS* associated with biological and psychosocial adversity [16]. Risk factors included male gender, low birth weight, poor/fair child health rating, poor maternal mental health, lower socioeconomic status (SES) and minority ethnicity. There was emerging evidence to suggest a dose response relationship between the number of risk factors and developmental risk on the *PEDS*. In addition, the greater the number of risk factors experienced by the child the more likely the child was to not have access to well child health services [17]. As such, the impact of biological and environmental risk factors on developmental outcomes and completion of developmental surveillance at well child checks will be examined in the WMG study birth cohort using a composite bio-ecological framework [18].

In this paper, development of the birth cohort of the WMG study is described, as are reasons for non-participation of eligible families in our cohort, their representativeness, the prevalence of risk factors known to be associated with poor developmental outcomes, and participant characteristics at six, 12, and 18-months follow-up. This will inform the applicability of the study findings for health service planning.

## Methods

### Study population

The WMG study was conducted in South West Sydney, which has seven local government areas (LGAs). It has a rapidly growing population with substantial cultural and linguistic diversity, and is characterised as having the accompanying health and psychosocial concerns of disadvantaged populations [19].

### Recruitment

Recruitment occurred between November 2011 and April 2013. In the initial phases of the WMG study, a pilot study was conducted through the child health nurses to assess their feasibility as primary recruiters. During the pilot study, child health nurses carried out home visits with new mothers within four weeks post-birth, and took on the recruitment role in terms of informing the mothers about the study. However, due to time constraints relating to their clinical role, and feeling unable to provide sufficient study information to obtain “informed consent”, they did not obtain their consent directly – instead, passing on the interested parents’ contact details to the research staff who then sent these parents information and consent forms. During the pilot, the response rate was low and so the alternative recruitment strategy of research staff approaching parents directly on postnatal wards was implemented.

The main recruitment settings were two postnatal wards in two public hospitals in South West Sydney. These two hospitals were selected from the four teaching hospitals in the area due to the high number of births and attendance by parents from culturally and linguistically diverse (CALD) backgrounds. Research staff attended the postnatal wards on a daily basis to recruit women who had recently given birth. They gave the new mothers (along with their partners, if available) information about the study. If parents indicated interest in taking part they gave them a detailed information sheet to read in addition to the written consent form. Recruitment documentation was available in Assyrian, Arabic, Vietnamese, Khmer, and Traditional Chinese, the main five non-English languages used by parents who gave birth at the hospitals. Written informed consent for participation in the study was obtained from the mothers (or father, if preferred). Parents, who declined to participate in the study when approached on the postnatal wards by research staff, were asked about the reasons for not wanting to participate.

### Ethics

Approval was obtained from the Human Research Ethics Committees of South Western Sydney Local Health District (SWSLHD) and the University of New South Wales to undertake the WMG study.

### Baseline measures

Baseline and follow-up risk factor measures collected in the WMG study cohort are outlined in Table 1 using the bio-ecological framework [18]. Data were self-reported by parents using baseline and 18-month follow-up questionnaires. These questionnaires included factors known to be important for child health and development that were derived from the extant literature and via an examination of questionnaires from other Australian cohort studies, such as the Longitudinal Study of Australian Children, [20, 21] and the Bulundidi Gudaga Study [22, 23]. Additional information routinely collected as part of the mothers’ antenatal and obstetric care was obtained through data linkage with electronic medical records (EMR). Socio-Economic Indexes for Areas (SEIFA) data for the families was also calculated using the suburb of residence. SEIFA constitutes a suite of indexes that rank geographic areas across Australia in terms of their socioeconomic characteristics based on five-yearly census data of people, families and dwellings within that area. A lower number denotes higher neighbourhood disadvantage [24].

**Table 1:** Baseline and follow-up measures

Risk measures	Instrument/Source	Birth	6 months	12 months	18 months
Child					
Gestational age, birth weight	EMR (birth)/Baseline survey	X			
Admission special care nursery (SCN) or Neonatal intensive care unit (NICU)	EMR (postnatal)	X			
Serious injuries/illness	18 month survey				X
General health, sleeping, feeding	18 month survey				X
Parental concerns indicating developmental risk	*Parents’ Evaluation of Developmental Status (PEDS)* [45]		X	X	X
Parent					
Maternal antenatal and postnatal health	EMR (antenatal screen), 18 month survey	X			X
Maternal Edinburgh Depression Scale (EDS)score > 12 [26]	EMR (antenatal screen)	X			
History of abuse in own childhood (mother)	EMR (antenatal screen)	X			
Smoking, alcohol use in pregnancy and postnatal	EMR (antenatal screen), 18 month survey	X			X
Breast feeding	NBQ/18 month survey	X			X
Maternal primary language	EMR (demographic)	X			X
Nationality	EMR (demographic)	X			
Country of birth	Baseline survey	X			
Maternal and paternal education, maternal and paternal employment	Baseline/18 month survey (LSAC adapted [20])	X			X
Cultural influences on parenting	18 month survey				X
Parenting	18 month survey				X
Stimulation (being read to)	18 month survey				X
Exposure to screen time	18 month survey				X
Access to toys	18 month survey				X
Family					
Annual Income	Baseline/18 month survey (LSAC adapted [20])	X			X
Income covers income covers living expenses	Baseline/18 month survey (Bulundidi Gudaga Study [22, 23])	X			X
Affordability of food, clothing, housing, energy, health care	18 month survey				X
Partner status (mother)	EMR (antenatal screen), 18 month survey	X			X
Family size	Baseline/18 month survey	X			X
Social support	Baseline/18 month survey (LSAC adapted [20])	X			X
Housing	18 month survey				X
Family history learning/mental/physical health problems	Baseline/18 month survey	X			X
Other children in out of home care	EMR (antenatal screen)	X			
History of being hit or slapped by partner in last 12 months (NSW Health Domestic Violence screening tool) [46]	EMR (antenatal screen)	X			
Family stressors					X
Neighbourhood					
SEIFA decile 1 [24]	EMR (demographic)	X			
Neighbourhood satisfaction	Baseline/18 month survey (LSAC [20])	X			X
Service Use					
Sources of information on early childhood development	Baseline survey, (LSAC adapted [20])	X			X
Attendance to health care	18 month survey				X
Difficulties with access to comprehensive health care	18 month survey				X
Satisfaction with health care	18 month survey				X

EMR electronic medical record, LSAC Longitudinal Study of Australian Children

### Outcome

At each six, 12 and 18-month follow-up, parents were contacted by phone and asked (through a standard questionnaire developed by the researchers) about attending well child checks for developmental surveillance. Key questions focused on whether they had taken their child for the recommended well child checks as outlined in their child’s personal health record (PHR), which health service(s) they used, their satisfaction level with that service, and whether a standardised screening tool (the *PEDS*) had been completed, by whom and what the results were [6]. At each follow-up call, the *PEDS* information in the PHR was collected. For those children where it was not documented in the PHR, parents were asked to complete the *PEDS* information with research staff over the phone. The *PEDS* is a parent-completed standardised questionnaire consisting of 10 items. It has been used to elicit parental concerns around child development for children aged less than eight years in populations, communities and clinical samples. The *PEDS* open-ended questions cover expressive and receptive language, fine motor skills, gross motor skills, behaviour, socialisation, self-care and learning [6]. An estimate of developmental risk as high, moderate, low or no risk is derived from the parental concerns recorded and then a clinical pathway is recommended. The *PEDS* has a sensitivity of 91-97 % and specificity of 73-86 % in recent validation studies from the United States for the accuracy of parental concerns in detecting children at high and/or moderate developmental risk [12].

### Analysis of representativeness and retention

EMR data from all infants born in a public hospital in SWSLHD during the study period, as well as their mother’s antenatal and obstetric clinical data, was exported from the SWSLHD medical records database. To establish the representativeness of the WMG cohort, WMG participant data (uniquely identified) was extracted from the main EMR dataset and this main dataset was subsequently used as a comparison. Representativeness was determined by Chi-square analyses of the available clinical, psychosocial and sociodemographic EMR data, categorised into bio-ecological levels of child, parent, family and neighbourhood, comparing the WMG participants to two groups: the population of birthing mothers/infants born in any of the public SWSLHD hospitals during the study period, and those born in two hospitals where recruitment of the WMG participants from the postnatal wards took place. Characteristics of the participants for whom there was *PEDS* data available at six, 12 and 18 months were compared with those participants who did not have *PEDS* data at each time point using Chi-square analyses.

### Analysis of baseline biological and environmental risk for future developmental risk

Descriptive frequencies and percentages are used in this paper to describe baseline characteristics and risk factors of the birth cohort. The proportion of infants exposed to multiple child, parent, household and neighbourhood risk factors available from baseline data in the WMG cohort and demonstrated in the recent systematic review to be associated with parental concerns indicating high developmental risk on the *PEDS* was examined [16]. At the *child level,* perinatal risk (defined as a child who was low birth weight (<2,500 g) and/or preterm (<37 weeks gestation) and/or had an admission to special care nursery or neonatal intensive care) was included. At the *parent level,* maternal Middle Eastern or Asian nationalities were included (in line with Australian Bureau of Statistics (ABS) coding) as they represented the two major minority groups in the local population [25]. At the *family level,* English not being the primary household language was included. At the *neighbourhood level,* a SEIFA score in the lowest decile was included [24]. Binary variables were created for each of the individual risk factors (0 absence, 1 presence) to give a possible range of 0–4. Poor maternal mental health (according to the Maternal Edinburgh Depression Scale Score >12 [26]) and family-level measures of socioeconomic disadvantage, such as annual household income and maternal education, were not able to be included because when these risk factors were included, complete data on all such risk factors were available for only 1211 participants (60 % of all baseline participants). All analyses were completed using STATA: Data Analysis and Statistical Software (STATA) version 13 [27].

## Results

### Cohort recruitment at baseline

Between November 2011 and April 2013, child health nurses forwarded the details of 785 infants to research staff. The parents of these infants had verbally agreed to be contacted by research staff. Of this group, 626 (80 %) of infants had parents who did not agree to participate, or could not be reached, or did not return consent forms. This left 159 (20 %) infants whose parents agreed to participate out of the total number of parents told of the study by the child health nurses.

**Fig. 1:**
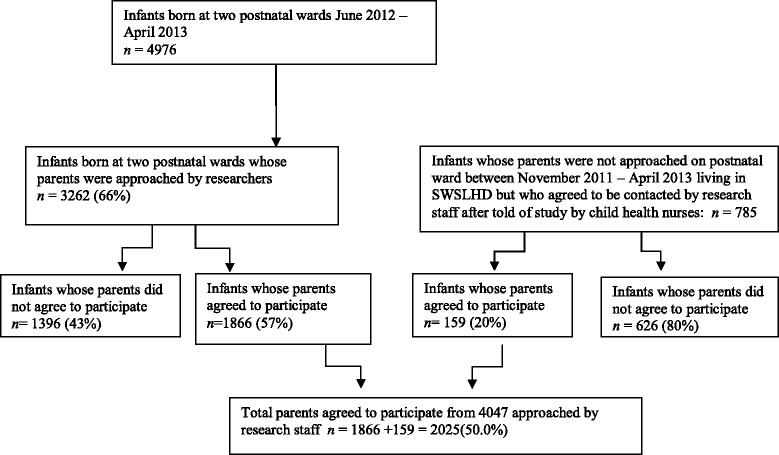
Recruitment numbers by method

During the study period of June 2012 to April 2013, research staff also approached parents of 3,262 (66 %) of the 4,976 infants born at the two hospitals during this period who were on the postnatal wards. Parents of 1,866 (57 %) of these infants agreed to participate. Thus of the 4,047 parents approached by the research team – either on the postnatal ward (3262) or through mail-outs after child health nurses passed on details to the research team (785), 2,025 (50 %) - 1866 through the postnatal wards and 159 through the child health nurse method - consented to participate (see Fig. 1).

Of note, in addition to the 1866 infants recruited through the postnatal ward of the two hospitals, 7 of the 159 infants recruited through the child health nurse method had attended the postnatal wards of the two hospitals between June 2012 to April 2013. These 1,873 infants made up 38 % of the total number of infants on the postnatal wards of the two hospitals for same period (*n* = 4,976).

The reasons for declining to participate were collected from 1370 (98 %) of the 1396 eligible parents who did not agree to participate from the two hospital postnatal wards. The main reasons given were: lack of interest (341 participants (25 %)); too busy (290 participants (21 %)); no reason given (176 participants (13 %)); undecided (172 participants (13 %)); language barriers (75 participants (6 %)); relocation (67 participants (5 %)); past/current research involvement (58 participants (4 %)); privacy concerns (57 participants (4 %)); husband would not agree (52 participants (4 %)); happy with current system (32 participants (2 %)); baby/mother unwell (28 participants (2 %)); too tired (14 participants (1 %)), and lack of access to a phone (8 participants (1 %)).

### Representativeness

Representativeness of the WMG study infants compared to infants from the two postnatal wards where direct recruitment occurred and all four hospitals in SWSLHD is described in Table 2. When WMG study infants were compared with infants from the two hospital postnatal wards who were *not* recruited to the study over the study period of November 2011 to April 2013: a significantly lower proportion of WMG infants were male (48 % versus 51 %, *p* = .014); less of their mothers had a primary language that was not English (23 % versus 27 %, *p* = .001), and more of their mothers had experienced abuse in their own childhoods (8 % versus 6 %, *p* = .008).

**Table 2:** “Watch Me Grow” cohort representativeness of the postnatal ward and SWSLHD non-participants #proportions based on available data

Characteristic	“Watch Me Grow” *N* = 2013 infants; *N* = 1976 mothers n (%) #	Non participants (two postnatal wards) *N* = 5540 infants; *N* = 5371 mothers n (%) #	Non participants (all South West Sydney) *N* = 12494 infants; *N* = 12208 mothers n (%) #
Child			
Male	964 (48.0)	2826 (51.2) *p* = .01	6431 (51.6) *p* = .002
Female	1047 (52.0)	2697 (48.8)	6024 (48.4)
Mean birth weight (g)	3291.6 (SD 590.7)	3281.2 (SD 618.9)	3349.4 (SD 565.3) *p* < .001
Low birth weight (<2500 g)	151 (7.5)	474 (8.6)	722 (5.8) *p* = .004
Mean gestational age (weeks)	38.8 (SD 2.0)	38.7 (SD 2.2)	38.9 (SD 1.9) *p* = .03
Preterm(<37 weeks)	180 (9.0)	498 (9.0)	860 (6.9) *p* = .009
Admitted SCHN/NICU	301 (15.0)	868 (15.8)	1423 (11.4) *p* < .001
Mother			
Mean maternal age (years)	30.1 (SD 5.5)	30.1 (SD 27.6)	29.7 (SD 18.8)
Maternal age < 20 years	48 (2.4)	115 (2.1)	321 (2.6)
Maternal smoking in pregnancy	87 (5.3)	265 (6.0)	661 (7.3) *p* = .005
Maternal alcohol during pregnancy	23 (1.3)	53 (1.1)	171 (1.6)
Antenatal health problems	621 (32.0)	1642 (33.8)	3068 (26.5) *p* < .001
Mother experienced child abuse as a child	126 (7.5)	256 (5.7) *p* = .008	795 (7.6)
Poor maternal mental health EDS >12	116 (7.1)	346 (7.7)	738 (7.1)
Family			
Primary language on antenatal visit	458 (23.2)	1427 (26.6) *p* = .003	2897 (23.8)
Mother Australian nationality	825 (41.7)	2283 (42.5)	6212 (50.9) *p* < .001
Mother Middle Eastern and Asian nationality	848 (42.2)	2262 (42.1)	4309 (35.3) *p* < .001
Mother has no partner at antenatal check	74 (4.0)	233 (4.9)	618 (5.7) *p* = .005
Hit, slapped, hurt by partner in last year	19 (1.1)	66 (1.4)	152 (1.4)
A child already in out-of-home care	41 (2.6)	124 (2.8)	317 (3.2)
Neighbourhood			
SEIFA decile 1	855 (44.2)	2417 (46.1)	4491 (37.5) *p* < .001

When WMG study infants were compared with infants born in all four hospitals in SWSLHD who were *not* recruited to the entire study period of November 2011 to April 2013: a significantly lower proportion of WMG infants were male (48 % versus 52 %, *p* = .002); however more WMG infants were preterm (9 % versus 7 %, *p* = .0009); low birth weight (8 % versus 6 %, *p* = .004) and/or admitted to the special care nursery (SCN) or neonatal intensive care (NICU) (15 % versus 11 %, *p* < .001). Less WMG infants had mothers who: had smoked in the second half of pregnancy (5 % versus 7 %, *p* = .003); were of Australian nationality (42 % versus 51 %, *p* < .001), and did not have a partner (4 % versus 6 %, *p* = .006). A significantly greater proportion of WMG infants had mothers with antenatal health problems (32 % versus 27 %, *p* < 001). A significantly greater proportion of the WMG infants came from households that were in the most disadvantaged decile on the SEIFA (44 % versus 38 % *p* < 001).

Additional baseline survey data was available for 1,761 (87 %) participants in the WMG birth cohort. Unfortunately, as it was not available in EMR, it was not able to be compared with eligible non-participants. The majority of WMG parents were born overseas (58 % of mothers and 61 % of fathers). At their antenatal check, 42 % mothers identified a nationality that was defined as Middle Eastern or Asian as per ABS coding [25]. For those mothers born overseas, the five top countries of birth were Vietnam (10 %), Lebanon (6 %), Iraq (4 %), New Zealand (3 %) and India (3 %). For those families speaking a language other than English, the main languages were Arabic (14 %), Vietnamese (9 %), Hindi (2 %), Bengali (2 %), Urdu (2 %) and traditional Chinese (1 %). In terms of education, income and neighbourhood disadvantage, 19 % of mothers had not completed the last two years of high school in NSW, and 15 % of households had an annual income less than AUD 25,001.

### Retention

Of the original 2,025 participants enrolled at birth, 792 (39 %) had six-month *PEDS* data, 649 (32 %) had 12-month *PEDS* data and 565 (28 %) had 18-month *PEDS* data (see Table 3). Overall, *PEDS* data was available for 1,034 participants at least at one time point in the six to 18-month follow-up period (51 % response rate), and 314 participants (16 %) had *PEDS* data available at all three points in time. Eighty three (4 %) participants withdrew from the study and 171 (8 %) were never contacted during the follow-up period.

**Table 3:** Characteristics of mothers and children at 6, 12, 18 months with *PEDS* outcome data collection at each follow-up compared to those who did not have outcome data collected (participant vs non-participant)

Characteristic	Baseline- birth *N* = 2013 n (%)	6 months with PEDS data *N* = 792 n (%)	12 months with PEDS data *N* = 649 n (%)	18 months with PEDS data *N* = 565 n (%)
Child Level				
Male gender	964 (48.0)	344 (46.5)	281 (46.1)	244 (44.9)
Low birth weight (<2500 g)	151 (7.5)	49 (6.6)	45 (7.4)	38 (7.0)
Preterm (<37 weeks)	180 (9)	57 (7.7)	57 (9.3)	51 (9.4)
Admitted SCHN/NICU	301 (15)	102 (13.8)	94 (15.4)	86 (15.8)
Parents				
Maternal age < 20 years	48 (2.4)	10 (1.4) *p* = .02	9 (1.5)	8 (1.5)
Maternal smoking in pregnancy	88 (5.2)	23 (3.7) = 0.03	15 (2.9) *p* = .005	11 (2.4) *p* = .001
Maternal alcohol during pregnancy	23 (1.3)	7 (1.0)	8 (1.4)	6 (1.2)
Antenatal health problems	640 (32.4)	230 (31.2)	196 (32.5)	182 (33.8)
Mother experienced child abuse as a child	128 (7.5)	45 (7.0)	41 (7.7)	34 (7.0)
Poor maternal mental health (EDS >12)	121 (7.3)	36 (6.0)	25 (5.1)	29 (6.5)
Mother did not complete high school	316 (18.5)	110 (14.5) *p* < 001	90 (14.4) *p* = .001	81 (14.6) *p* = .005
Family				
English not primary language on antenatal visit	463 (23.0)	167 (22.6)	147 (24.1)	128 (23.6)
Mother Australian nationality	846 (42.5)	323 (43.6)	271 (44.4)	233 (42.9)
Mother Middle Eastern or Asian nationality	848 (42.2)	307 (41.5)	249 (40.8)	226 (41.6)
Annual income at birth < AUD25001	277 (17.6)	94 (13.2) p < 001	75 (12.9) *p* < 001	60 (11.8) *p* < 001
Mother has no partner at antenatal check	74 (4.0)	16 (2.4) *p* = .004	14 (2.5) *p* = .02	14 (2.8)
Hit, slapped, hurt by partner in last year	19 (1.1)	7 (1.1)	6 (1.1)	4 (0.8)
A child already in out-of-home care	42 (2.7)	9 (1.4) *p* = .01	3 (0.6) *p* < 001	2 (0.4) *p* < 001
Neighbourhood				
SEIFA decile 1	872 (44.2)	274 (37.3) *p* < 001	221 (36.5) *p* < 001	200 (37.0) *p* < 001

Infants who had *PEDS* data collected at six months were significantly less likely to have mothers who: were aged under 20 years (*p* = .02); smoked during pregnancy (*p* = .03);were single (*p* = .005); did not complete high school (*p* < 001); and/or have a sibling in out-of-home care (*p* = .02); and/or have an annual household income < AUD 25,001 (*p* < 001), and/or reside in a disadvantaged neighbourhood (lowest SEIFA decile) (*p* < 001) when compared with those who did not have *PEDS* data collected at six months.

Infants who had *PEDS* data collected at 12 months were significantly less likely to have mothers who: smoked during pregnancy (*p* = .005); were single (*p* = .02); did not complete high school (*p* = .001); and/or have a sibling in out-of-home care (*p* < 001); and/or have an annual household income < AUD 25,001 (*p* < 001); and/or reside in a disadvantaged neighbourhood (lowest SEIFA decile) (*p* < 001) compared with those who did not have *PEDS* data collected at 12 months.

Infants who had *PEDS* data collected at 18 months were significantly less likely to have mothers who: smoked during pregnancy (*p* = .001); did not complete high school (*p* = .005); and/or have a sibling in out-of-home care (*p* < 001); and/or have an annual household income < AUD 25,001(*p* < 001); and/or reside in a disadvantaged neighbourhood (lowest SEIFA decile) (*p* < 001) compared with those who did not have *PEDS* data collected at 18 months.

### Number of baseline risk factors for future developmental risk

The proportion of infants with the risk factors of: perinatal risk (low birth weight, and/or preterm and/or admission to the SCN/NICU); maternal Middle Eastern or Asian nationality; English not being the primary household language; and/or neighbourhood SEIFA score in the lowest decile, were examined. Of these, 691 (35 %) WMG infants were exposed to one risk factor, 451 (23 %) were exposed to two, 268 (14 %) were exposed to three, and 34 (2 %) were exposed to four risk factors.

## Discussion

In addition to experiencing inequities in health and health care, people experiencing socioeconomic disadvantage and/or who are from CALD backgrounds are less likely to participate in research [15]. Thus, there is an “inverse research law” – with those who stand to benefit most from population and health services research being under-represented so that their needs go unmeasured and views unheard [28]. The WMG study had an overall participation rate of 50 % of participants approached, with 38 % of those potentially being eligible. Although this participation rate is lower than most other large scale birth cohorts, [15, 29] the WMG birth cohort goes some way to address inequity in research by having a cohort that is broadly representative of the local CALD population. This is vital for the applicability of the WMG study in understanding a whole-of-population approach to developmental surveillance. However, even within this birth cohort, there is still participation bias. There is greater participation by parents with English as their primary language. At follow-up, participants in the baseline cohort deemed to be at psychosocial risk were more likely to not have *PEDS* outcome data available.

The WMG cohort has significantly greater representation by infants who were preterm, low birth weight, admitted to the SCN or NICU and having a mother with poorer antenatal health compared to non-participants in SWSLHD. This may be a reflection of the fact that one of the recruiting hospitals has a NICU and there are more opportunities to recruit a family if they are in hospital for longer. This is a strength of the WMG cohort because in the literature, these biological risk factors are associated with adverse developmental outcomes; thus, the engagement of these groups in investigating barriers to developmental surveillance is valuable [29].

For effective recruitment into longitudinal studies, it is critical that the health professionals and the end users are enlisted to help recruit participants. In the initial phases of the WMG study, child health nurses took on the recruitment role by informing mothers about the study. This approach however, resulted in low recruitment rates – presumably due to the extra steps parents of a newborn infant would need to take in having to return consent forms by post or online. In contrast, when researchers directly approached parents of newborn infants in the postnatal wards there was greater participation. The opportunity to discuss the study objectives directly with the participants and the provision of the consent form at the same time seem to have enhanced the recruitment rate. However, recruiting in the immediate postnatal period means that one is still trying to engage parents at the time a new infant enters a family’s life. On reflection, the addition of prenatal and/or antenatal recruitment may have improved the overall participation rate, but with a person-power cost.

We have useful information on the reasons for declining to participate from eligible families. The same reasons have been demonstrated to be barriers to research participation in other observational studies, both in Australia and internationally [29–32]. Research into non-participation has also postulated that the increasing demands on the population in general to take part in market surveys and research projects, the perceived increasing complexity of research and a general decline in volunteerism in the community, may play a role [33]. For this study, cultural factors such as barriers to knowledge regarding the importance of early childhood development and community attitudes to identifying children with developmental problems, may also influence participation [34, 35]. Although we did not exclude families with poor English proficiency and we had research documents translated into the key languages of the community, the lack of bilingual researchers may have contributed to language barriers being given as a reason for non-participation. The under-representation of parents whose primary language was not English in the WMG study birth cohort is thus not surprising.

With regards to cohort follow-up, there were significant challenges in collecting *PEDS* outcome data at the six, 12 and 18-month follow-up. Barriers to this included frequent changes in phone numbers and also having to make numerous attempts for successful phone contact which necessitated significant person-power resources. Although our baseline cohort was representative of the population it sampled, at each of the follow-up periods, we were less likely to collect data from those mothers and infants at greater psychosocial risk, thereby introducing differential participation in the follow-up component of our study. Pleasingly, there was no differential participation found for those mothers from diverse cultural backgrounds and non-English speaking households in the collection of *PEDS* outcomes at six, 12 and 18 month follow-up groups.

When one examines the baseline risk factors for developmental risk of the WMG cohort through a bio-ecological lens, 39 % of children were exposed to at least two risk factors associated with an increase in a child’s risk of having developmental problems [17, 36–40]. Many risk factors that increase the risk of developmental problems (including socioeconomic disadvantage, minority ethnicity and language barriers) also increase the risk of not accessing primary health care services [41–43]. It is reasonable to postulate that our prospective follow- up will demonstrate significant associations between at least some of these risk factors with developmental risk and not accessing developmental surveillance services.

### Strengths and limitations

An important strength of this study is the ability to link routinely collected participant EMR data with the study data. This has provided a clear picture of the extent to which the WMG cohort is representative, and highlights any potential biases. It has provided data without overburdening parents of recruited children, and has also allowed prospectively collected comprehensive data on psychosocial and biological risk factors in the antenatal and perinatal period to be made available for analysis, even though this is a birth recruitment cohort. In addition, it allows for a comprehensive analysis of representativeness of the cohort with comparative data on an extensive range of risk factors between participants, and eligible non-participants. The main limitation with the EMR data is that we only have directly comparable area deprivation measures using SEIFA, which is not a family or individual measure of socioeconomic disadvantage. This may impact on the assessment of representativeness and baseline risk. In addition, there was minimal paternal data available in EMR for the antenatal or perinatal period. Given that the WMG cohort is broadly representative of mothers and infants attending the postnatal wards from which they were recruited, it would be reasonable to postulate that the household income, employment and educational levels are similar to the eligible non-participants for participating mothers and fathers. A significant limitation is the differential participation at follow-up for families and their infants at greater psychosocial risk. This may impact on the power of the study in being able to analyse the impact of psychosocial risk factors on study outcomes and the ability to generalise our findings.

## Conclusion

The “Watch Me Grow” study has been designed to provide Australian evidence on the barriers and facilitators to early identification of children at risk of developmental disorders in a culturally, linguistically and socioeconomically diverse population. Children from families that are socially disadvantaged and/or are of CALD backgrounds may be more at risk of adverse developmental outcomes and inequitable access to health services such as developmental surveillance, and are also the least likely to participate in research [14, 15, 44]. Recruitment in the WMG study has resulted in a birth cohort that is over represented by families of CALD backgrounds and groups at biological risk through inclusive and even preferential recruitment in an attempt to redress this inequity in research participation. In the follow-up of this cohort, representation by families of CALD backgrounds has been maintained despite substantial loss to follow-up. It is envisaged that the WMG study findings will provide important evidence to support the development of leading practice in early identification of developmental disorders for all children and their families.

## Abbreviations

ABS: Australian Bureau of Statistics
CALD: culturally and linguistically diverse
EMR: electronic medical record
LSAC: Longitudinal Survey of Australian Children
NICU: neonatal intensive care
NSW: New South Wales
PEDS: Parents’ Evaluation of Developmental Status (PEDS)
SCN: special care nursery
SEIFA: Socio-Economic Indexes for Areas
SWSLHD: South West Sydney Local Health District
WMG: “Watch Me Grow” study
